# PReS-FINAL-2228: Survey of off-label ANTI-IL1 treatments in France: two years data

**DOI:** 10.1186/1546-0096-11-S2-P218

**Published:** 2013-12-05

**Authors:** L Rossi-Semerano, B Fautrel, D Wendling, E Hachulla, C Galeotti, A Meyer, S Ottaviani, R Dhote, O Richer, M Fouillet-Desjonqueres, I Touitou, I Koné-Paut

**Affiliations:** 1 Department of Paediatrics and Paediatric Rheumatology, Hôpital de Bicêtre, National Reference Centre for Auto-inflammatory Diseases, Le Kremlin Bicêtre, France; 2 Departement of Rheumatology, Hôpital La Pitié Salpetrière, Université Pierre et Marie Curie, Paris, France; 3 Department of Rheumatology, CHU Besançon, Besançon, France; 4 Department of Internal Medicine, National Reference Center for Rare Autoimmune and Systemic Diseases, Lille, France; 5 Department of Rheumatology, Hôpitaux de Strasbourg, Strasbourg, France; 6 Department of Rheumatology, CHU Bichat, Paris, France; 7 Department of Rheumatology, CHU Avicenne, Bobigny, France; 8 Department of Pediatrics, CHU Bordeaux, Bordeaux, France; 9 Department of Paediatrics and Paediatric Nephrology, Hôpital femme mère enfant, Lyon, France; 10 Unité Médicale des Maladies Auto-Inflammatoires, Laboratoire de Génétique, Hôpital A de Villeneuve, CHRU Montpellier, Montpellier, France

## Introduction

Despite their limited licensed indications, anti-IL1 agents are often used in real-life practice for an increasing number of diseases. A national survey to record the off-label use of this class of therapeutics in France was started in January 2011. The survey is coordinated by the French National Reference Centre for Auto-inflammatory Diseases, under the aegis of the "Club Rhumatisme et Inflammation".

## Objectives

The survey aims at gathering information concerning: the number of patients treated with anti-IL1 agents in France, the treated disease, the kind and the indication of the used anti-IL1 agents, their efficacy and safety.

## Methods

We set up a physician-directed questionnaire covering the following areas: patient data, disease data, anti-IL1 agent (molecule, dose and frequency), its efficacy, adverse events. Any adult or paediatric patient who had received an anti-IL1 agent from January 2005 in France could be included.

## Results

At two years 193 patients from 37 centres have been included. Demographic data: 104 males, 89 females; 141 adult, 52 paediatric patients, mean age 35.2 years at treatment onset. Main diseases were: adult onset Still disease (AoSD) (35), systemic onset juvenile idiopathic arthritis (SoJIA) (29), gout (27), anakinra-treated CAPS (22), mevalonate kinase deficiency (MKD) (14), familial Mediterranean fever (FMF) (12), SAPHO syndrome (9), Schnitzler's syndrome (7). The main off-label used agent was anakinra, used at least once in 189 patients. Canakinumab was used in 25 patients, mainly children, in most cases as a second-line treatment after anakinra. Rilonacept is not yet available in France. 83 patients (66 anakinra, 17 canakinumab treated patients) were still on treatment at last visit. Some form of clinical response was found in 90% of anakinra-treated patients. A complete physician-evaluated response was reported in Schnitzler's syndrome (85%), gout (80%), CAPS (75%), AoSD (59%), FMF (50%), SoJIA (42%), MKD (30%), SAPHO (11%). 83% of canakinumab-treated patients showed clinical response. At least one adverse event (AE) was reported for 53% and a serious adverse event (SAE) for 10% of anakinra treated patients. Main AEs were: injection site reactions (48%), weight gain (11%) and liver enzymes elevation (9%). SAEs were principally severe infections, macrophage activation syndrome and severe hepato-toxicity. 50% of patients treated with canakinumab showed an AE, namely respiratory infections and liver anomalies. Only few patients had a SAE (severe infections).

## Conclusion

Anakinra is the main off-label anti-IL1 agent used in France, showing partial to complete efficacy in most patients; complete clinical response rates vary according to specific diseases, being higher in Schnitlzler syndrome, gout, CAPS and AoSD. Around half of the patients showed at least one AE, mainly related to a poor local tolerance. Preliminary data of our survey suggest that canakinumab was efficacy and well tolerate in most patients.

## Disclosure of interest

L. Rossi-Semerano: None Declared, B. Fautrel: None Declared, D. Wendling: None Declared, E. Hachulla Consultant for: SOBI Biovitrum, Novartis, C. Galeotti: None Declared, A. Meyer: None Declared, S. Ottaviani: None Declared, R. Dhote: None Declared, O. Richer: None Declared, M. Fouillet-Desjonqueres: None Declared, I. Touitou: None Declared, I. Koné-Paut Grant/Research Support from: SOBI Biovitrum, Consultant for: Novartis.

